# Community-Reported Postvaccination Experiences and Their Influence on COVID-19 Vaccine Uptake and Acceptance in Rural Southwestern Uganda: A Qualitative Cross-Sectional Study

**DOI:** 10.7759/cureus.111860

**Published:** 2026-07-01

**Authors:** Jonans Tusiimire, Ronus Ajuna, Gershom Muganga, Shallot Tumuhairwe, Miriam J Nakiwala, Martha K Namakula, Marvel E Komukyeya, Daniel C Mwandah

**Affiliations:** 1 Department of Pharmacy, Faculty of Health Sciences, Mbarara University of Science and Technology, Mbarara, UGA; 2 Department of Pharmaceutical Sciences, Faculty of Health Sciences, Mbarara University of Science and Technology, Mbarara, UGA; 3 Department of Pharmacology and Toxicology, School of Pharmacy, Kampala International University Western Campus, Kampala, UGA

**Keywords:** adverse effects, covid-19 vaccines, southwestern uganda, vaccine hesitancy, vaccine uptake

## Abstract

Background: The COVID-19 pandemic had profound global health and socioeconomic impacts, necessitating rapid vaccine deployment to reduce morbidity and mortality. However, perceptions regarding vaccine safety have influenced vaccine confidence and uptake in many communities. This study explored community-reported experiences of adverse events following COVID-19 vaccination and their implications for vaccine acceptance, uptake, and public perceptions in Mitooma District, Southwestern Uganda.

Methods: A qualitative cross-sectional study was conducted using focus group discussions (FGDs) among community members and leaders from seven randomly selected villages in Katenga Subcounty, Mitooma District, Southwestern Uganda. Participants were purposively selected based on their involvement, influence, or knowledge of COVID-19 vaccination-related issues. Data were collected on reported experiences following vaccination, perceptions regarding vaccine safety, and factors influencing vaccine acceptance and uptake.

Results: A total of 52 participants (male-to-female ratio, 1:1) participated in the FGDs. The median age (range) of participants was 45 (23-82) years. Most participants were Banyankole (n = 45, 86.5%), married (n = 46, 88.5%), and engaged in subsistence farming (n = 26, 50%). More than half (n = 28, 53.8%) had attained primary education as their highest level of education. Participants commonly reported experiencing symptoms after COVID-19 vaccination, ranging from mild and expected local or systemic reactions to severe or unusual experiences that they perceived as vaccine-related. The most frequently reported experiences included injection-site symptoms, fever, dizziness, and gastrointestinal symptoms. Some participants also described rare and alarming events, including perceived nerve-related problems, impotence, and deaths occurring after vaccination; however, these reports were based on participant accounts and were not clinically verified or confirmed to be causally related to COVID-19 vaccination. Concerns regarding reported adverse events, amplified by misinformation and anti-vaccine messages in local media, negatively influenced willingness among some individuals to receive additional vaccine doses. Nevertheless, overall vaccine acceptance and uptake remained high, largely attributed to fear of severe COVID-19 outcomes and previous positive experiences with immunization programs.

Conclusion and recommendations: Community perceptions of vaccine safety were shaped by both personal experiences and information circulating within communities. Engagement of community leaders and participatory approaches were important in supporting vaccination efforts. Strengthening pharmacovigilance systems, improving mechanisms for reporting and evaluating adverse events following immunization, and implementing proactive risk communication strategies are essential for addressing vaccine safety concerns and maintaining public trust.

## Introduction

The COVID-19 pandemic caused unprecedented global health, social, and economic disruptions. Since the emergence of SARS-CoV-2 in late 2019, the pandemic has resulted in substantial morbidity and mortality worldwide. By March 2024, the World Health Organization (WHO) had reported more than 774 million confirmed COVID-19 cases and over seven million officially reported deaths globally, although the true burden was likely higher due to variations in testing capacity, reporting systems, and excess mortality estimates [1]. In Africa, more than 13 million confirmed cases and approximately 258,000 deaths had been reported, while Uganda recorded over 170,000 confirmed cases and more than 3,600 deaths [1,2].

Before vaccines became available, countries implemented a range of nonpharmaceutical interventions (NPIs), including lockdowns, movement restrictions, physical distancing, masking, and limitations on public gatherings, to reduce SARS-CoV-2 transmission and prevent overwhelming healthcare systems. Although these measures contributed to controlling transmission, they also resulted in significant economic losses, disrupted education, affected livelihoods, and increased social inequalities, particularly in low- and middle-income countries (LMICs) [3,4].

The rapid development and authorization of COVID-19 vaccines represented a major milestone in pandemic response. Several vaccine platforms, including messenger RNA (mRNA), viral vector, and inactivated virus technologies, demonstrated substantial efficacy in reducing symptomatic infection, severe disease, hospitalization, and COVID-19-related mortality [5,6]. Although vaccines did not completely prevent SARS-CoV-2 transmission, widespread vaccination enabled countries to progressively relax public health restrictions, restore social and economic activities, and reduce pressure on health systems.

In Uganda, the Ministry of Health (MoH) launched the national COVID-19 vaccination campaign in March 2021 following receipt of vaccines through the global COVAX facility and other bilateral and multilateral vaccine access mechanisms. The initial phase of the vaccination program prioritized frontline healthcare workers, teachers, security personnel, older adults, and individuals with underlying medical conditions because of their increased risk of exposure and severe COVID-19 outcomes [7,8]. As vaccine availability increased, eligibility was expanded to include the wider population, including adolescents aged 12 years and above in accordance with national vaccination guidelines [7].

Between March 2021 and June 2022, Uganda administered several COVID-19 vaccine products, including AstraZeneca/Oxford, Pfizer-BioNTech, Moderna, Janssen (Johnson & Johnson), Sinopharm, and Sinovac vaccines [7]. These vaccines employed different technological platforms, including viral vector vaccines (AstraZeneca and Janssen), nucleic acid-based vaccines (Pfizer-BioNTech and Moderna), and inactivated virus vaccines (Sinopharm and Sinovac). Emergency use authorization by regulatory authorities facilitated rapid deployment during the public health emergency; however, vaccine safety monitoring remained essential because phase I-III clinical trials, while providing critical evidence on efficacy and common adverse effects, were conducted within relatively limited populations and follow-up periods [9,10].

The primary objectives of COVID-19 vaccination programs were to reduce COVID-19-related mortality and morbidity, prevent severe disease, protect healthcare systems, and facilitate socioeconomic recovery. Vaccination remains one of the most effective public health interventions for controlling infectious disease outbreaks [11]. However, the effectiveness of vaccination programs depends not only on vaccine availability but also on public confidence, acceptance, and uptake. Studies conducted across LMICs have demonstrated considerable variability in COVID-19 vaccine acceptance, with reported acceptance rates ranging widely across populations and settings [6,12].

Despite increased vaccine availability, vaccine uptake remained suboptimal in many settings, including Uganda. By the end of November 2022, approximately 41.2% of Uganda’s population had received at least one COVID-19 vaccine dose, while 27.4% were fully vaccinated [7]. Low uptake has been associated with multiple demand-side and supply-side factors, including concerns regarding vaccine safety and adverse effects, misinformation, inadequate knowledge, mistrust of health authorities and vaccine manufacturers, and limited access to vaccination services [13-15].

A scoping review of COVID-19 vaccine hesitancy in Africa identified vaccine safety concerns, fear of adverse events, misinformation, and distrust in vaccine manufacturers and health authorities as common barriers to vaccine acceptance [13]. Similarly, studies conducted in Uganda reported that concerns about vaccine safety, uncertainty regarding vaccine effectiveness, inadequate information, and low perceived risk of COVID-19 infection contributed to hesitancy and reduced uptake [14,16,17]. Importantly, the drivers of vaccine hesitancy vary according to social, cultural, geographic, and health-system contexts, highlighting the need for locally generated evidence to inform tailored interventions.

Understanding adverse effects associated with COVID-19 vaccines and how these experiences influence public perceptions is particularly important because concerns about vaccine safety can strongly affect acceptance of current and future vaccination programs. Therefore, this study was conducted in Mitooma District, Southwestern Uganda, to examine COVID-19 vaccine-related adverse effects and their implications for vaccine acceptance, uptake, and public perceptions. The findings provide locally contextualized evidence to guide targeted risk communication strategies, strengthen vaccine confidence, and inform preparedness for future vaccination campaigns and public health emergencies.

## Materials and methods

Study design and setting

**Figure 1:**
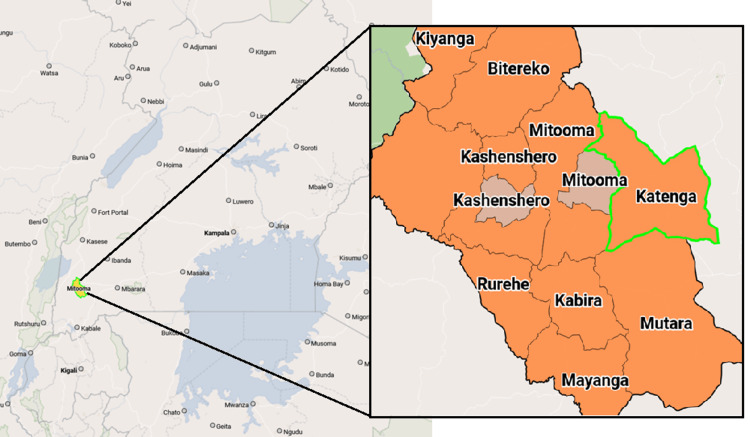
Map of Uganda showing the location of Mitooma District which has been expanded to reveal the constituent sub-counties, including Katenga (study area) This map has been reproduced with minor modifications from the Uganda Bureau of Statistics [19,20]. The copyright states that "The contents from www.ubos.org may freely be reproduced, provided that Uganda Bureau of Statistics is cited as source. The source referenced must include the link to point in question on ubos.org where the figures can be viewed in their full context by the user" and can be accessed here: https://www.ubos.org/about-us/guidelines-on-usage-and-reproduction-of-data/?utm_source

A qualitative cross-sectional study was conducted between January and March 2024 in Katenga Subcounty, Mitooma District, Southwestern Uganda (Figure 1). Mitooma District was established in 2010 following its separation from Bushenyi District and became fully operational on July 1, 2010. According to the Uganda National Housing and Population Census 2024, the district has a population of 226,009, a land area of 544.0 km², and a population density of 415.5 persons per km², with females constituting 55.7% of the population [18]. The district comprises one county (Ruhinda), three constituencies, eleven subcounties, and five town councils. The health system includes one Health Center IV, six Health Center IIIs, and eight Health Center IIs [19]. Katenga Subcounty was selected because it represents a typical rural setting where COVID-19 vaccination services were implemented, allowing exploration of community-level perceptions and experiences.

Study population

The target population comprised residents of Katenga Subcounty who had received COVID-19 vaccination and whose experiences and perceptions could inform understanding of reported postvaccination events, vaccine safety perceptions, and uptake behaviors. The study population included residents and key stakeholders within Katenga Subcounty representing village-level social structures (Local Council I units). The village-based Local Council 1 (LC I) was considered the primary unit of inquiry because vaccination experiences and perceptions were expected to vary across villages. The study therefore included vaccinated residents as well as influential community actors such as LC I chairpersons, village health team (VHT) members, women leaders, church leaders, and other knowledgeable residents.

Sampling strategy

A two-stage sampling approach was used. First, seven villages were randomly selected from Katenga Subcounty (Table 1). Second, participants were purposively selected within each village in collaboration with village mobilisers based on their knowledge, lived experience, and involvement in vaccination-related community activities.

**Table 1:** The villages and participant numbers for the FGDs in Katenga, Mitooma District. Two FGDs were conducted in both Katenga and Ngoma FGDs: focus group discussions

FGD No.	Village	Number of participants
1	Katenga I	7
2	Katenga II	7
3	Bitooma I	9
4	Rwagashani	8
5	Nyaruzinga I	9
6	Ngomba I	6
7	Ngoma II	6
Total		52

Focus group discussions (FGDs) and data collection procedures

Seven FGDs were conducted, one in each selected village (LC I), with each FGD representing the village as the unit of community inquiry. Each FGD comprised 6-9 participants, yielding a total sample size of 52 participants.

A semistructured interview guide developed from literature on COVID-19 vaccine uptake in Uganda and other LMICs was used [6,12,14,15,17,21]. The guide contained five sections covering sociodemographic characteristics, pandemic experiences, vaccine knowledge and attitudes, barriers to uptake, and community-based strategies for improving vaccine acceptance. The guide was developed in English, translated into Runyankole, and pretested in a neighboring subcounty not included in the study.

All FGDs were conducted in Runyankole by trained research assistants. Data collection was carried out by six research assistants and supervised by three senior researchers. The assistants were organized into three teams, each consisting of a moderator and a notetaker/audio recorder.

Data collection was completed over two days. On day 1, three FGDs were conducted concurrently by the three teams. On day 2, three FGDs were again conducted concurrently, after which one additional FGD was conducted by a rotated team to complete the seventh village. Each team conducted no more than one FGD per day to minimize fatigue and maintain consistency in facilitation quality.

FGDs were held at designated village venues commonly used for community meetings. Venues were identified with village mobilizers and community leaders and approved by the research team to ensure accessibility and appropriateness.

Data analysis

Audio recordings were transcribed verbatim by two members of the research team fluent in Runyankole and English. Transcripts were cross-checked against audio recordings for accuracy and completeness. Where translation into English was required, meaning was carefully preserved through iterative review by the research team.

Data were analyzed using manifest qualitative content analysis. This approach was selected because the study aimed to systematically describe and organize participants’ explicitly stated experiences, perceptions, and views regarding COVID-19 vaccination rather than interpret underlying meanings.

Two investigators independently read and re-read the transcripts to become familiar with the data. Meaningful units of text were identified and assigned initial codes using both deductive categories derived from the study objectives and inductive codes emerging from the data. The researchers compared coding outputs, discussed discrepancies, and reached consensus on a final coding framework. Codes with similar meanings were grouped into categories, which were subsequently abstracted into themes. Themes were reviewed iteratively to ensure coherence and alignment with the dataset and study objectives.

To enhance trustworthiness, investigator triangulation, audit trails, and verbatim quotations were used. Credibility was strengthened through independent coding and consensus discussions. Dependability was supported by systematic documentation of analytical decisions. Confirmability was ensured through continuous comparison of findings with raw data, while transferability was enhanced by detailed description of the study setting, sampling approach, and participant characteristics.

Ethical considerations

Ethical approval was obtained from the Mbarara University of Science and Technology Research and Ethics Committee (MUST-2024-1390), and administrative clearance was obtained from the Mitooma District Health Office. Written informed consent was obtained from all participants.

Confidentiality and voluntary participation were emphasized, and participants could withdraw at any time without consequence. Given the village-based FGD structure, care was taken to minimize social pressure through neutral facilitation and assurance of confidentiality. All audio recordings and field notes were securely stored and accessed only by the research team for research purposes.

## Results

Participants’ demographics

A total of 52 participants (male-to-female ratio: 1:1) were involved in the FGDs. The median age (range) of the participants was 45 (23-82) years. The majority (n = 45, 86.5%) were Banyankole, married (n = 46, 88.5%), with four (range: 0-15) children and practicing subsistence farming (n = 26, 50%). More than half (n = 28, 53.8%) had stopped their education at the primary level (Table 2).

**Table 2:** Participants’ demographic characteristics

Characteristics	Frequencies, N (%)
Gender	
Male	26 (50.0)
Female	26 (50.0)
Age in years (median, range)	45 (23-82)
Religion	
Catholic	26 (50)
Anglican	24 (46.2)
Others	2 (3.8)
Marital status	
Married	46 (88.5)
Single	4 (7.7)
Divorced	2 (3.8)
Occupation	
Farmer	26 (50)
Peasant	8 (15.4)
Housewife	1 (1.9)
Teacher	2 (3.8)
Others	15 (28.8)
Education level	
None	1 (1.9)
Primary	28 (53.8)
Secondary	17 (32.7)
Tertiary	6 (11.5)
Tribe	
Munyankole	45 (86.5)
Other	7 (13.5)
Number of children, median (range)	4 (0-15)
Number of children	
None	5 (9.6)
3 Jan	13 (25.0)
6 Apr	21 (40.4)
8 Jul	9 (17.3)
>8	4 (7.7)

Nature of reported postvaccination experiences

Table 3 summarizes participants’ reported experiences following COVID-19 vaccination as described during focus group discussions, reflecting community perceptions and narratives. These accounts were organized into 12 thematic categories, representing a broad range of perceived postvaccination health experiences.

**Table 3:** Self-reported adverse effects following COVID-19 vaccination that came up during FGDs FGDs: focus group discussions All the events reported in the above table were based on participants’ self-reported experiences during FGDs and reflect community perceptions and narratives following COVID-19 vaccination. The accounts were not clinically verified, nor was causality between vaccination and the reported events established or inferred

S/N	Themes	Codes
1	Respiratory issues	Difficulty in breathing
2	Feeling sick after getting the vaccine	Feeling sick
3	Dizziness	Feeling dizzy
4	Unexplained negative effects	Vaccine treating them badly
5	General body weakness	Body weakness
6	Arm-related side effects	-Pain at site of injection
		-Inability to raise arm
		-Inability to move the arm
		-Arm paralysis
		-Arm swelling
		-Arm weakness
7	Fever	Fever
8	Gastrointestinal effects	-Vomiting
		-Diarrhea
9	Reproductive health-related side effects	-Low libido
		-Impotence
		-Amenorrhea
10	Insomnia	Failure to sleep
11	Nerve-related side effects	-Nerve damage
		-Nerve pain
		-Lower body paralysis
12	Rare and peculiar side effects	-Hair loss
		-Fungal infection
		-Death

The most frequently reported experiences involved localized arm-related symptoms, particularly pain at the injection site, swelling, heaviness, and temporary restricted movement. Some participants also described more persistent functional limitations affecting daily activities, including prolonged weakness or reduced mobility of the affected arm.

“I received the vaccine but after receiving it, my whole arm became very heavy as if paralyzed as if it wanted to come off the body piece by piece.” (MITFG001)

In addition to localized symptoms, participants reported a range of systemic symptoms that they perceived to occur following vaccination, including fever (lasting up to three days), dizziness, vomiting, general body weakness, fainting, diarrhea, and short-term respiratory discomfort. These were generally described as temporary and variable in intensity across individuals.

Some participants further described less common and more severe or unusual health events, including nerve-related symptoms, hair loss, insomnia, and gastrointestinal disturbances. These accounts reflected community narratives and perceptions of more atypical postvaccination experiences.

“What happened to me is that the nerve in my arm died and even the nerve that transports to the head.” (MITFG004)

A small number of participants also reported concerns related to reproductive health, including menstrual irregularities and impotence. In addition, isolated accounts included fungal infections and deaths reported within communities in temporal proximity to vaccination. These reports were based on participant narratives and were not clinically verified.

“Some women failed to get their menses back.” (MITFG004)

Participants indicated variability in the perceived onset and duration of these experiences, ranging from transient symptoms lasting minutes or hours to longer-lasting symptoms persisting for weeks or months. Some participants also perceived more pronounced reactions following second doses or after receiving different vaccine types; however, these observations were based on subjective comparisons within community experiences rather than verified clinical assessments.

Perceived influence of reported postvaccination experiences on vaccine uptake and acceptance

Table 4 presents themes describing how participants perceived that reported postvaccination experiences influenced vaccine uptake, acceptance, and community perceptions in Mitooma District. Three broad themes were identified: emergence of negative sentiments towards vaccination, reinforcement of misconceptions and uncertainties, and concerns related to livelihood and daily functioning.

**Table 4:** Key themes and examples showing the reported side effects of COVID-19 vaccines

Themes	Created negative feelings	Fueled misconceptions	Livelihood concerns
Codes	Fear of COVID-19 vaccine, hatred for the vaccine, discouragement of others	Fuel for misconceptions about vaccines	Economic uncertainties (work disruptions, loss of income)

Participants described that reported postvaccination experiences contributed to increased negative sentiments towards COVID-19 vaccination within some community members. These included fear of vaccination, reduced willingness to receive subsequent doses, and discouragement of others from getting vaccinated, largely based on shared community narratives and observed or heard experiences.

Participants also reported that such experiences reinforced existing misconceptions and uncertainties about vaccine safety. These perceptions were shaped by community discussions, informal information exchange, and interpretation of temporally associated health events following vaccination.

In addition, participants noted that concerns about potential disruption of daily activities and income generation influenced vaccine hesitancy. Some community members reportedly feared that postvaccination symptoms could interfere with work, particularly for individuals engaged in subsistence farming and informal labor, thereby affecting household livelihoods.

In some participants, the perceived severity and duration of reported postvaccination experiences were associated with strong negative emotional responses toward COVID-19 vaccination, including rejection and reduced willingness to receive further doses.

 “I received two doses that affected me for two whole weeks, so I hate it so much now. You can’t take me back.” (MITFG001)

In other cases, participants reported that observing or hearing about similar experiences among peers contributed to fear, hesitation, and reduced confidence in vaccination.

“They feared, seeing how someone else responded and the side effects they got.” (MITFG004)

Such community observations were described as reinforcing existing doubts, uncertainties, and misconceptions about COVID-19 vaccination, particularly in contexts where participants perceived limited access to clear or trusted counter-information from health authorities.

Perceived lessons for vaccine safety communication and monitoring

Participants described community engagement as an important facilitator of COVID-19 vaccination uptake and trust within Katenga Subcounty. The involvement of local leaders and VHT members was perceived to strengthen community confidence and responsiveness by bridging communication between health authorities and community members.

“I was a teacher as a VHT. It was my obligation so I went first so that when I am telling people I am their example... Though when I went and I received it, I announced to my people and they all went and received, and it did not have any issue." (MITFG001)

Participants further noted that such leadership involvement helped to promote public health measures during the pandemic, including hygiene practices and preventive behaviors, which were perceived to support broader COVID-19 control efforts at the community level.

“Another thing when the chairman invited us, they kept teaching us to constantly wash our hands, boil drinking water, and clean the food that we were going to eat...” (MITFG005)

Effective communication was described as essential in shaping community perceptions of COVID-19 vaccination. Participants emphasized that timely, clear, and culturally appropriate messaging helped to build understanding and confidence, whereas perceived gaps in communication or conflicting messages contributed to uncertainty and doubt.

Some participants reported that exposure to divergent messages from different sources, including public media and perceived expert opinions, created confusion regarding vaccine safety and efficacy. In such contexts, participants indicated that uncertainty was sometimes reinforced when conflicting narratives were not adequately addressed through trusted communication channels.

“Concerning vaccination, we are getting some confusion between academicians and the government… When people are in that confusion, they conclude that… maybe the government has realized that what he/she is saying is the truth…” (MITFG001)

Participants also reported limited awareness of formal vaccine safety monitoring systems, including pharmacovigilance and postvaccination surveillance mechanisms. This lack of visibility was perceived to reduce confidence in the systems responsible for monitoring vaccine safety and responding to potential adverse events, thereby influencing trust in vaccination programs at the community level.

## Discussion

Nature of reported postvaccination experiences and perceptions of vaccine safety

This study explored community-reported experiences following COVID-19 vaccination and how these experiences shaped perceptions of vaccine safety in a rural Ugandan setting. Participants commonly described localized symptoms such as injection-site pain, swelling, soreness, and heaviness, as well as systemic symptoms including fever, fatigue, headache, chills, myalgia, dizziness, and gastrointestinal discomfort. These experiences are consistent with commonly recognized short-term reactions following vaccination, which reflect expected immune responses and typically resolve within a few days, as documented by the WHO and the United States Centers for Disease Control and Prevention (CDC) [9,22].

In addition to these commonly reported reactions, participants described less common and more severe health events, including paralysis-like symptoms, neurological complaints, reproductive health concerns, hair loss, fungal infections, and deaths occurring after vaccination. These accounts represent community perceptions and narratives rather than clinically verified adverse events, and no causal relationship between vaccination and the reported outcomes can be established from this study. Nevertheless, the findings highlight the importance of understanding how communities interpret health events occurring after vaccination. Perceived associations between vaccination and subsequent illness may influence public confidence irrespective of whether a causal relationship exists. Such interpretations may be shaped by attribution bias, recall bias, coincidental illness, and exposure to conflicting information, particularly in settings where access to trusted health information and formal mechanisms for adverse event assessment is limited [23].

Perceived influence of reported postvaccination experiences on vaccine uptake, acceptance, and perceptions

The findings suggest that community interpretations of reported postvaccination experiences influenced attitudes towards COVID-19 vaccination. Participants described fear, anxiety, uncertainty, and reduced trust among individuals who believed that vaccination had negatively affected their health. Some reported reluctance toward receiving subsequent doses and, in certain cases, discouragement of others from vaccination. These findings are consistent with evidence showing that concerns about vaccine safety and fear of adverse events are important determinants of vaccine confidence and acceptance, particularly in low-resource settings [6].

Participants also described how reported health events reinforced existing concerns and uncertainties surrounding COVID-19 vaccination. Reproductive health concerns, for example, were interpreted by some community members as supporting fears that vaccines could affect fertility. This reflects broader processes of risk perception whereby individuals may interpret new information through pre-existing beliefs and concerns, including confirmation bias [24,25]. In closely connected rural communities, such interpretations can spread rapidly through social networks and shape collective perceptions of vaccine safety.

Economic considerations also emerged as an important influence on vaccine perceptions. Participants, particularly those engaged in subsistence farming and other physically demanding occupations, expressed concerns that symptoms such as fatigue, weakness, or muscle pain could interfere with their ability to work and earn income. Similar findings have been reported elsewhere in Uganda, where concerns about disruption of daily activities and loss of income were identified as barriers to vaccine acceptance [14]. Together, these findings suggest that vaccine decision-making is influenced not only by perceptions of safety but also by the anticipated social and economic consequences of vaccination.

Community engagement and risk communication

An important finding from this study was the central role of community engagement in shaping vaccine confidence. VHTs and local leaders were perceived as trusted sources of information who promoted vaccine uptake by serving as role models and facilitating communication between health services and communities. This observation is consistent with evidence from other settings demonstrating that trusted community actors can positively influence vaccine confidence and acceptance, particularly in resource-constrained environments [26-28].

However, participants also described exposure to conflicting messages regarding vaccine safety through public communication channels, including radio and informal community networks. The influence of these messages appeared to be strengthened by the perceived credibility of those delivering them. In contexts where distinguishing between scientific evidence, personal opinion, and unverified claims may be challenging, conflicting narratives can contribute to uncertainty and affect perceptions of vaccine safety. Similar challenges have been documented globally, where delayed, inconsistent, or unclear communication has been associated with reduced vaccine confidence and increased hesitancy [29]. These findings underscore the importance of timely, transparent, and culturally appropriate risk communication strategies that directly address community concerns and emerging narratives.

Lessons for vaccine safety monitoring

The study also identified limited awareness and visibility of formal vaccine safety monitoring mechanisms at the community level. Participants reported that concerns about postvaccination experiences were often communicated informally to VHTs or healthcare workers rather than through recognized adverse events following immunization (AEFI) reporting pathways. Although this study did not evaluate the effectiveness of pharmacovigilance systems, the findings suggest potential opportunities to strengthen community awareness of vaccine safety monitoring processes and improve integration between community-level reporting structures and formal surveillance systems.

Strengthening these linkages may facilitate earlier identification and assessment of vaccine safety concerns, improve feedback to communities, and enhance trust in immunization programs. Given that perceptions of vaccine safety are often shaped by community narratives and lived experiences, effective pharmacovigilance should extend beyond technical surveillance systems to include active community engagement, responsive communication, and visible mechanisms for addressing public concerns [30].

Strengths and limitations of the study

This study provides valuable insights into community experiences and perceptions regarding COVID-19 vaccination in a rural Ugandan setting, contributing evidence from an underrepresented context. The inclusion of participants from diverse village-level social structures, including community leaders, VHTs, and residents, allowed exploration of varied perspectives on vaccination experiences and vaccine confidence. FGDs enabled rich contextual data and collective reflection on how vaccine-related narratives are formed and shared within communities.

However, several limitations should be noted. Data were collected in 2024, several years after the initial vaccination rollout, introducing potential recall bias in participants’ accounts. In addition, social desirability bias and group dynamics may have influenced responses during FGDs. The purposive sampling approach, while appropriate for selecting information-rich participants, may limit representativeness. The study also lacked triangulation with healthcare providers, clinical records, or vaccination registers; therefore, self-reported experiences could not be independently verified and should be interpreted as community narratives rather than confirmed clinical outcomes. Finally, findings are context-specific to Katenga Subcounty and should be interpreted cautiously when considering transferability to other settings.

## Conclusions

This study highlights the importance of understanding community perceptions and narratives surrounding postvaccination experiences in shaping COVID-19 vaccine confidence and acceptance in rural Uganda. While participants reported a range of experiences following vaccination, these accounts were not clinically verified and should not be interpreted as confirmed vaccine-related adverse effects. However, the findings demonstrate that perceived associations between vaccination and subsequent health events can influence trust, willingness to receive vaccines, and community recommendations. Strengthening vaccine confidence will require timely, transparent, and culturally appropriate risk communication, alongside improved community awareness and engagement with vaccine safety monitoring systems. Enhanced collaboration between the MoH, the Uganda National Drug Authority (NDA), health workers, VHTs, and local leaders is essential to strengthen pharmacovigilance, address emerging concerns, counter misinformation, and sustain public trust in immunization programs.

## Appendices

Informed consent form and questionnaire

A focus group interview guide for a study to establish the barriers to COVID-19 vaccine uptake in rural South Western Uganda

We are conducting a study to establish the barriers to, and facilitators for, COVID-19 vaccine uptake in rural South Western Uganda. This study will provide valuable insights on what community-based interventions the government should undertake so as to increase general vaccine acceptance and uptake by the population.

If you agree to participate, you will be placed in a group of 10 people: 8 from within your community, a facilitator, and a notetaker who will use an audiotape recorder to record participant responses. The discussion will take about 2-3 hours. Participants, as well as the facilitators, have signed a nondisclosure agreement to ensure that all information shared within the discussion remains confidential, and code names will be used throughout the discussion to maintain anonymity. All the documents as well as the tape recorder will be access-controlled.

Involvement in this study is voluntary, and you are free to withdraw from the study at any time. 

Thank you for your cooperation.

Declaration

I have understood the information about this study and my queries have been answered satisfactorily.

Participant’s code……………………………..

Participant’s signature …………………………

Date ……………………………………………

Section A: Demographic information

1. What is your

a)     Gender (ori omushaija nari omukazi?) ……………………………………

b)    Date of birth (okazaarwa ryari?) ..………...………………………………

c)     Religion (ediini yaawe) …...………………………………………………

d)    Marital status (obufumbo) ……………..…………………………………

e)     Occupation (omurimo)……….…..……………………………………….

f)     Level of education (okushoma kwawe) ………………………………….

g)    Tribe (oruganda) ………….……………………………………………...

h)    Number of Children (oine abaana bangahi?) …………………………….

i)      Place of residence (n’otuura nkahi?).…………………………………….

Section B: General information

Setting the Mood

1.     What do you know about the COVID-19 pandemic, and where did it find you?

2.     Briefly, what was your experience like during this pandemic? 

Did they or anyone close to them suffer from COVID-19?

What remedies did they or anyone close to them use to treat COVID-19?

How was their community affected by the pandemic?

What collective efforts as a community were made to mitigate its spread? (probe for individual contribution based on cadre)

Section C: COVID-19 vaccine knowledge and attitudes knowledge

3. One of the interventions that were put in place to mitigate the spread of COVID-19 by the Ministry of Health was through vaccination.

a) What do you know about vaccination in general? (P*robe for any myths, perceived benefits for both adults and children, sources of information on vaccination*)

b) How did they know about the COVID-19 vaccine and what was their reference in case they needed to know more about it? (*Probe for medical personnel, VHTs, social media, TVs, etc*.)

c) Were any of them vaccinated against COVID-19, or do they know of someone close to them that got vaccinated?

i. What do they think prompted them or people close to them to get vaccinated?

ii. Which vaccines did they receive and what side effects did they experience?

iii. Overall, would they recommend someone to get the COVID-19 vaccine? If yes, (a).* Why would they recommend it? (b). Do the same reasons apply to other vaccines* (*Probe for childhood immunization, perceived differences in benefits*?)

iv. If not, why? (*Probe for any myths, misconceptions, or concerns*)

d) What reasons do they or someone close to them give for not receiving the COVID-19 vaccine? (*Probe for any myths or misconceptions*)

i. Overall, would they recommend someone get the COVID-19 vaccine? *If yes,* (a). Why would they recommend it?; (b). Do the same reasons apply to other vaccines (*Probe for childhood immunization, perceived differences in benefits*?) 

ii. If not, why? (*Probe for any myths, misconceptions, or concerns*)

Section D: Barriers to COVID-19 vaccine uptake

4. What factors influenced their decision to receive or decline the COVID-19 vaccine?

5. What do they think are the main barriers that prevented people from getting vaccinated against COVID-19 within their community? (Probe for any other challenges within the community not mentioned above)

Section E: Community-based participatory appreciative inquiry intervention

6. What actions do they think could be taken to increase general vaccine uptake within their community? (Probe for COVID-19 vaccine, childhood immunisation)

i. Did they hear about any community-based interventions for increasing COVID-19 vaccine acceptance and uptake within their community? (Probe for mobile vaccination clinics, community health worker education/outreach, peer education, public awareness campaigns (e.g. radio, TV, billboards, incentives such as food, cash, prizes))

ii. Which of those community-based interventions do they consider to be the most effective and why?

iii. How can they be translated to improve vaccine uptake in general (Probe for effectiveness in childhood immunisation, other vaccines against the possible barriers)

***Thank you for your participation***
